# *NPC1* silent variant induces skipping of exon 11 (p.V562V) and unfolded protein response was found in a specific Niemann‐Pick type C patient

**DOI:** 10.1002/mgg3.1451

**Published:** 2020-09-15

**Authors:** Marisa Encarnação, Maria Francisca Coutinho, Soo Min Cho, Maria Teresa Cardoso, Isaura Ribeiro, Paulo Chaves, Juliana Inês Santos, Dulce Quelhas, Lúcia Lacerda, Elisa Leão Teles, Anthony H. Futerman, Laura Vilarinho, Sandra Alves

**Affiliations:** ^1^ Research & Development Unit Human Genetics Department National Institute of Health Doutor Ricardo Jorge Porto Portugal; ^2^ Newborn Screening Metabolism & Genetics Unit Human Genetics Department National Institute of Health Doutor Ricardo Jorge Porto Portugal; ^3^ Center for the Study of Animal Science CECA‐ICETA University of Porto Porto Portugal; ^4^ Department of Biomolecular Sciences Weizmann Institute of Science Rehovot Israel; ^5^ Centro de Referência de Doenças Metabólicas do Centro Hospitalar Universitário São João Porto Portugal; ^6^ Unidade de Bioquímica Genética Centro de Genética Médica Jacinto Magalhães ‐ Centro Hospitalar e Universitário do Porto (CHP) Porto Portugal; ^7^ Clinical and Experimental Human Genomics group (CEHG) UMIB‐Unit for Multidisciplinary Research in Biomedicine ICBAS University of Porto Porto Portugal; ^8^ MetabERN‐European Reference Network for Rare Hereditary Metabolic Disorder Reference Centre for Diagnosis and Treatment ‐ CHP Porto Portugal

**Keywords:** exon skipping, Niemann‐Pick type C, *NPC1*, RNA‐seq, silent variant, unfolded protein response

## Abstract

**Background:**

Niemann‐Pick type C (NPC, MIM #257220) is a neuro‐visceral disease, caused predominantly by pathogenic variants in the *NPC1* gene. Here we studied patients with clinical diagnosis of NPC but inconclusive results regarding the molecular analysis.

**Methods:**

We used a Next‐Generation Sequencing (NGS)‐panel followed by cDNA analysis. Latter, we used massively parallel single‐cell RNA‐seq (MARS‐Seq) to address gene profiling changes and finally the effect of different variants on the protein and cellular levels.

**Results:**

We identified novel variants and cDNA analysis allowed us to establish the functional effect of a silent variant, previously reported as a polymorphism. We demonstrated that this variant induces the skipping of exon 11 leading to a premature stop codon and identified it in NPC patients from two unrelated families. MARS‐Seq analysis showed that a number of upregulated genes were related to the unfolded protein response (UPR) and endoplasmic reticulum (ER) stress in one specific patient. Also, for all analyzed variants, the NPC1 protein was partially retained in the ER.

**Conclusion:**

We showed that the *NPC1* silent polymorphism (p.V562V) is a disease‐causing variant in NPC and that the UPR is upregulated in an NPC patient.

## Graphical abstract

We identified an NPC1 silent variant inducing skipping of exon 11 (p.V562V) and unfolded protein response was found in a specific Niemann‐Pick type C patient

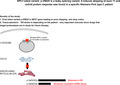

## 1 INTRODUCTION

Niemann‐Pick type C (NPC, MIM #257220) is an autosomal recessive lysosomal storage disease (LSD) with varying age of onset and progression (Patterson, 2007). One of its hallmarks is the intracellular accumulation of unesterified cholesterol and other lipids in various tissues (Lloyd‐Evans & Platt, 2010). NPC disease also affects the central nervous system (CNS) with the major clinical symptoms being vertical supranuclear gaze palsy (VSGP), cognitive impairment, ataxia, dystonia, dysarthria and/or dysphagia and progressive neurological deterioration (Garver et al., 2010; Patterson et al., 2017; Wijburg et al., 2012). The vast majority (~95%) of NPC patients have variants in the *NPC1* gene (MIM #607623) but in a few cases, the defective gene is *NPC2* (MIM #601015) (Carstea et al., 1997; Millat et al., 1999; Vanier, 1996). The *NPC1* gene encodes for NPC1, a transmembrane protein, which localizes in late endosomes (LE) and lysosomes (Naureckiene et al., 2000). It is highly glycosylated and its biosynthesis and trafficking pathways include co‐translational *N*‐glycosylation and correct folding in the endoplasmic reticulum (ER), transport to the Golgi apparatus and processing to a mature complex glycosylated protein and finally targeting to the lysosomes (Alfalah, Jacob, & Naim, 2002; Watari et al., 1999). NPC1 protein together with NPC2 (a small luminal lysosomal protein encoded by the *NPC2* gene) coordinate the transport of cholesterol out of the LE/lysosome compartment (Vanier, 2010). NPC1 is also essential for Ebola virus infection, having been described as its intracellular receptor (Gong et al., 2016). Recent works also indicate that NPC1 interacts with an ER‐resident protein and both are required for low‐density lipoprotein (LDL)—cholesterol egress from endocytic compartments to the ER (Du et al., 2011; Phillips & Voeltz, 2016; Raiborg, Wenzel, & Stenmark, 2015; Van Der Kant & Neefjes, 2014).

Over 400 mutations have been found in the *NPC1* gene (Shammas, Kuech, Rizk, Das, & Naim, 2019), the majority of them encode missense alleles (Schultz, Krus, & Lieberman, 2016). In recent years, a number of *NPC1* missense pathogenic variants have been categorized with regard to their trafficking ability and it was shown that most of them result in conformational changes disrupting NPC1 protein trafficking to the lysosome (Shammas et al., 2019). As a consequence, the misfolded mutants are retained in the ER and can trigger ER‐association degradation (ERAD) and unfolded protein response (UPR). The UPR along with the heat‐shock responses (HSR) are cytoprotective for the toxic effects of misfolded protein aggregates (Cox & Cachón‐González, 2012). UPR activation has been reported in other LSDs (Cox & Cachón‐González, 2012), namely, in patients with neuropathic Gaucher's disease. For this disease in particular there are reports on UPR activation caused not only by missense mutations that generate misfolded protein but also by a small insertion that causes a premature stop codon (PTC). This last observation suggests that small peptides can also activate the UPR pathway (Maor et al., 2013, 2016).

In the study reported here, we started with an NGS‐targeted DNA sequencing approach in two patients presenting with a clinical NPC suspicion. We identified compound heterozygosity between a novel putative missense variant (p.V505G) and a silent variant (p.V562V; previously reported as benign) (Fernandez‐Valero et al., 2005) in an individual with the juvenile onset of the neurological disease. After conducting appropriate molecular studies, we established the functional effect of the silent p.V562V in the *NPC1* splicing process, and also found it in two NPC patients from a second unrelated family. To get insights into further pathomechanisms in patient P1, we conducted a transcriptomics approach and found that genes that were differentially expressed in this patient (compound heterozygous for p.V505G/V562V), compared to other patients and controls, were associated with the UPR and ER stress. Interestingly, in all patients’ skin fibroblasts analyzed (most of them harboring missense mutations), we observed that the trafficking of the mutated NPC1 protein was affected.

## 2 MATERIALS AND METHODS

### 2.1 Ethical compliance

The studies were conducted in agreement with the Declaration of Helsinki and approved by the Ethics Committee of Instituto Nacional de Saúde Dr. Ricardo Jorge (2015DGH1062 and 2016DGH1312).

### 2.2 Patients

In total, our sample included five unrelated NPC patients and one affected sibling, all suffering from the juvenile form of the disease. Two of those patients had a clinical suspicion of the disease but had not been molecularly classified (Group I, Table 1), while all the others had already been analyzed for the *NPC1* gene in a previous study (Ribeiro et al., 2001) and were recalled for further analysis.

**TABLE 1:** Summary of biochemical, clinical, and molecular characteristics of the studied patients

		Patient	Biochemical Phenotype (Filipin)	Clinical subtype	Age at neurological onset (first symptoms)	Mutation	Protein	Location	Region affected	Reference
Group I										
Patients analyzed by ngs	Family 1 (F1) Portuguese	P1	Classical	Juvenile	6 years	c. 1514T>G c.1686G=A	p.V505G/V562V^a^	Ex9/Ex11	Luminal loop (between TM2 and TM3)	This work/(Fernandez‐Valero et al., 2005)
	Tunisian Patient	P2	n.a.	n.a.	n.a.	c.7G=A/ c.2882A=G	p.A3T/N961S	Ex1/Ex19	N‐terminus/Cysteine‐rich luminal loop (between TM‐8 and TM‐9)	(Runz et al., 2008)/(Dvorakova et al., 2006)
Group II										
Revisited patients	Family 2 (F2) Portuguese	P3 (F2:1)	Variant	Juvenile	7 years	c.1552C=T c.1686G=A	p.R518W/V562V^a^	Ex9/Ex11	Luminal loop (between TM‐2 and TM‐3)	(Ribeiro et al., 2001)/This work
		P3′ (F2:2)	Variant	Juvenile	7 years	c.1552C=T c.1686G=A	p.R518W/V562V^a^	Ex9/Ex11	Luminal loop (between TM‐2 and TM‐3)	(Ribeiro et al., 2001)/This work
Group III										
NPC patients (previously diagnosed) used in mars seq analysis as npc controls		P4	Variant	Juvenile	7 years	c.2932C=T c.3662delT	p.R978C/F1221fsX20	Ex20/Ex24	Cysteine‐rich luminal loop (between TM‐8 and TM‐9)/TM‐13; C‐terminus	(Ribeiro et al., 2001)
		P5	Classical	Juvenile	7 years	c.1552C=T c.3104C=T	p.R518W/A1035V	Ex9/Ex21	Luminal loop (between TM‐2 and TM‐3) Cysteine‐rich luminal loop (between TM‐8 and TM‐9)	(Ribeiro et al., 2001)

Note:

Patients P3 and P3′ are siblings.

GeneBank references: *NPC1*: NM_000271.5, Transcript ENST00000269228.10].

Abbreviations: Ex, exon; F, family; n.a., information not available; P, patient; TM, transmembrane.

^a^ Reclassified as a disease‐causing mutation (leads to exon skipping and premature stop codon).

The two original patients, who lacked molecular characterization, were included in a training set for a custom‐targeted NGS‐panel for LSD, composed of a group of 30 patients with clinical and/or biochemical diagnosis of LSD. Later, a third patient and her affected sibling, who had already been molecularly screened for NPC, were also enrolled in the study. These two individuals were revisited in a targeted cDNA analysis to screen for the effect of the silent variant, previously classified as a polymorphism. We also recruited samples from two additional NPC patients, who had a previous clinical (juvenile form) and molecular diagnosis of NPC (Ribeiro et al., 2001). These latter patient samples were also included in the transcriptomic analysis. Informed consent for gene research was obtained from individual or their guardians.

### 2.3 NGS‐targeted gene panel

Genomic DNA was extracted from peripheral blood and 50 ng was used for the library preparation according to the manufacturer's protocol (SureSelect Capture Library, Agilent). Genes of interest (exons and flanking regions) were captured with the SureSelect QXT kit followed by sequencing on the Illumina MiSeq platform according to the manufacturer's protocol for paired‐end 150 bp reads. The MiSeq Reporter Software (Illumina) was used for sample demultiplexing and FASTQ file generation. The software SureCall (Agilent) was used for alignment and variant calling and wANNOVAR for variant annotation. The candidate mutations were inspected visually using SureCall genome viewer to exclude false‐positive results and later confirmed by Sanger sequencing. Parental studies were conducted to ensure allele segregation in both families for whom variants in the *NPC1* gene [NM_000271.5, Transcript ENST00000269228.10] were identified and both genomic DNA and cDNA were analyzed in the parents. For cDNA analysis, RNA extracted from skin fibroblasts and/or blood was reverse‐transcribed and analyzed.

### 2.4 Cell culture

Primary fibroblasts derived from patients and controls were cultured in high‐glucose DMEM (Life Technologies, Carlsbad, CA) supplemented with 10% FBS, 1% penicillin/streptomycin at 37 °C and 5% CO_2._ Primary fibroblasts were used between passages 3 and 10.

### 2.5 Cycloheximide treatment

Cycloheximide (CHX) treatment was conducted as follows: patient P1 and control fibroblasts were seeded in 6‐well plates (400,000 cells/well) and 24 h after plating, the cells were treated with different concentrations of CHX (Sigma‐Aldrich, St. Louis, MO): 0.75 mg/ml; 1.5 mg/ml and 2 mg/ml. DMSO was used as a negative control. Next, 5 h after treatment, the cells were collected, total RNA was isolated, and cDNA synthesized for subsequent amplification as described below.

### 2.6 Reverse transcription PCR amplification and quantitative real‐time PCR

Total cellular RNA was isolated from cultured fibroblasts by extraction with the GRS Total RNA Kit (GRiSP, Porto, Portugal) or RNeasy Plus Micro (QIAGEN, Hilden, Germany). Total RNA from blood was collected into a PAXgene Blood RNA Tube (BD) and prepared using the PAXgene Blood RNA Kit (QIAGEN, Hilden, Germany). Total RNA (between 250 ng and 5 µg) was reverse transcribed to cDNA using Ready‐to‐go You‐Prime First‐Strand beads (GE Healthcare, Marlborough, MA) or the qScript cDNA Synthesis Kit (Quantabio, Beverly, MA). cDNA was amplified for regular PCR or real‐time PCR using either ImmoMix™ Red (Bioline, London, UK) or SYBR Green Master Mix (Bio‐Rad, Hercules, CA), and template‐specific primers (available upon request). Relative quantification of gene expression was performed using the comparative threshold (Ct) method as described by the manufacturer. Changes in mRNA expression level were calculated following normalization to *CTNNA1* gene expression (unchanged gene in MARS‐Seq). For data analysis, the ∆∆Ct method (Livak & Schmittgen, 2001) was used; fold‐changes were calculated as the difference in gene expression between control cells and NPC patient cells. Prediction of splicing alterations was performed with the software EX‐SKIP (https://ex‐skip.img.cas.cz/) and the Human Splicing Finder (HSF), an online bioinformatics tool to predict splicing signals (https://www.genomnis.com/access‐hsf).

### 2.7 MARS‐seq

We used MARS‐seq for the generation of RNA‐Seq libraries. RNA‐Seq libraries were sequenced using the Illumina NextSeq‐500, raw reads were mapped to the genome (GRCh38/hg38) using HISAT (version 0.1.6) and only reads with unique mapping were considered for further analysis. With corrected reads, we analyzed using BBCU‐NGS pipelines supplied by the Bioinformatics unit in the Weizmann Institute of Science. Normalization of the counts and differential expression analysis was performed using DESeq2 (Love, Huber, & Anders, 2014). Raw P values were adjusted for multiple testing using the procedure of Benjamini and Hochberg (Hochberg, 1988).

### 2.8 Enzymatic deglycosylation and western blot (WB)

Cells were homogenized in lysis buffer (50 mM Tris‐HCl pH = 8.0, 150 mM NaCl, 2 mM EDTA, 0.5% Nonidet P‐40 supplemented with proteinase inhibitors), resuspended and centrifuged at 1000 *g* for 10 min at 4°C. The supernatants’ protein contents were quantified, separated on 4%–12% precast gels Bis‐Tris (NuPAGE, Invitrogen, Carlsbad, CA) subjected to SDS‐PAGE (20 µg of total protein) and semi‐dry transferred to nitrocellulose membranes (Whatman, GE Healthcare, Marlborough, MA). For enzymatic deglycosylation of proteins, cell extracts were incubated in the presence or absence of 1 unit of PNGase F and Endo H (NEB, Ipswich, MA) for 1 h at 37°C according to the manufacturer's instructions. NPC1 protein was detected by incubation with anti‐NPC1 rabbit polyclonal antibody (ab108921, Abcam, Burlingame, CA) overnight and horseradish peroxidase (HRP)—conjugated IgG as the secondary antibody (sc 2313, Santa Cruz Technologies, Santa Cruz, CA). Signal was developed using enhanced chemiluminescence (ECL) (GE Healthcare, Marlborough, MA) and visualized on a ChemiDoc™ XRS^+^ (Bio‐Rad, Hercules, CA). Band intensity was calculated in Image Lab software version 6.0 (Bio‐Rad, Hercules, CA), after background subtraction and are expressed as Endo H‐sensitive/Endo H‐resistant ratios.

### 2.9 Statistics

GraphPad Prism 5.0b was used to determine statistical significance. One‐way ANOVA with Tukey or Bonferroni post hoc analysis were used as indicated in the figure legends. All error bars are SEM.

### 2.10 Confocal and fluorescence microscopy

Controls and patients’ skin fibroblasts were plated/seeded in µ‐slide 8‐well chambers (Nunc, Roskilde, Denmark) fixed with 4% PFA/PBS for 30 min, quenched with 0.05 M NH_4_Cl, permeabilized in ice‐cold methanol, and blocked with 5% BSA (Sigma‐Aldrich, St. Louis, MO) according to standard procedures. Primary antibodies used were: mouse anti‐LAMP1 (1:200, sc‐2001; Santa Cruz Biotechnology, Santa Cruz, CA) rabbit anti‐NPC1 (1:500, ab1089921, Abcam, Burlingame, CA) and goat anti‐Calnexin (1:200 dilution, AB0041, Sicgen, Cantanhede, Portugal). Secondary antibodies were labeled with Alexa Fluor 488 or 555 (1:1000, Thermo Fisher Scientific, Waltham, MA). The coverslips were washed three times in PBS and once in water, mounted (Fluoroshield^TM^ with DAPI, Sigma‐Aldrich, St. Louis, MO) and examined by fluorescence microscopy (Leica DM 4000B microscope). The images were acquired on a Leica TCS‐SPE confocal microscope and spectral detection adjusted for the emission of DAPI, Alexa 488, and Alexa 555 fluorochromes using the 405‐, 488‐, and 546‐laser lines, respectively. Digital images were analyzed using ImageJ version 2.0.0.

The method used for the evaluation of cellular cholesterol by filipin staining was performed as described (Vanier et al., 1991). The images were acquired on a fluorescence Nikon Eclipse E400 microscope and quantified using ImageJ version 2.0.0.

## 3 RESULTS

### 3.1 Molecular genetics

**FIGURE 1:**
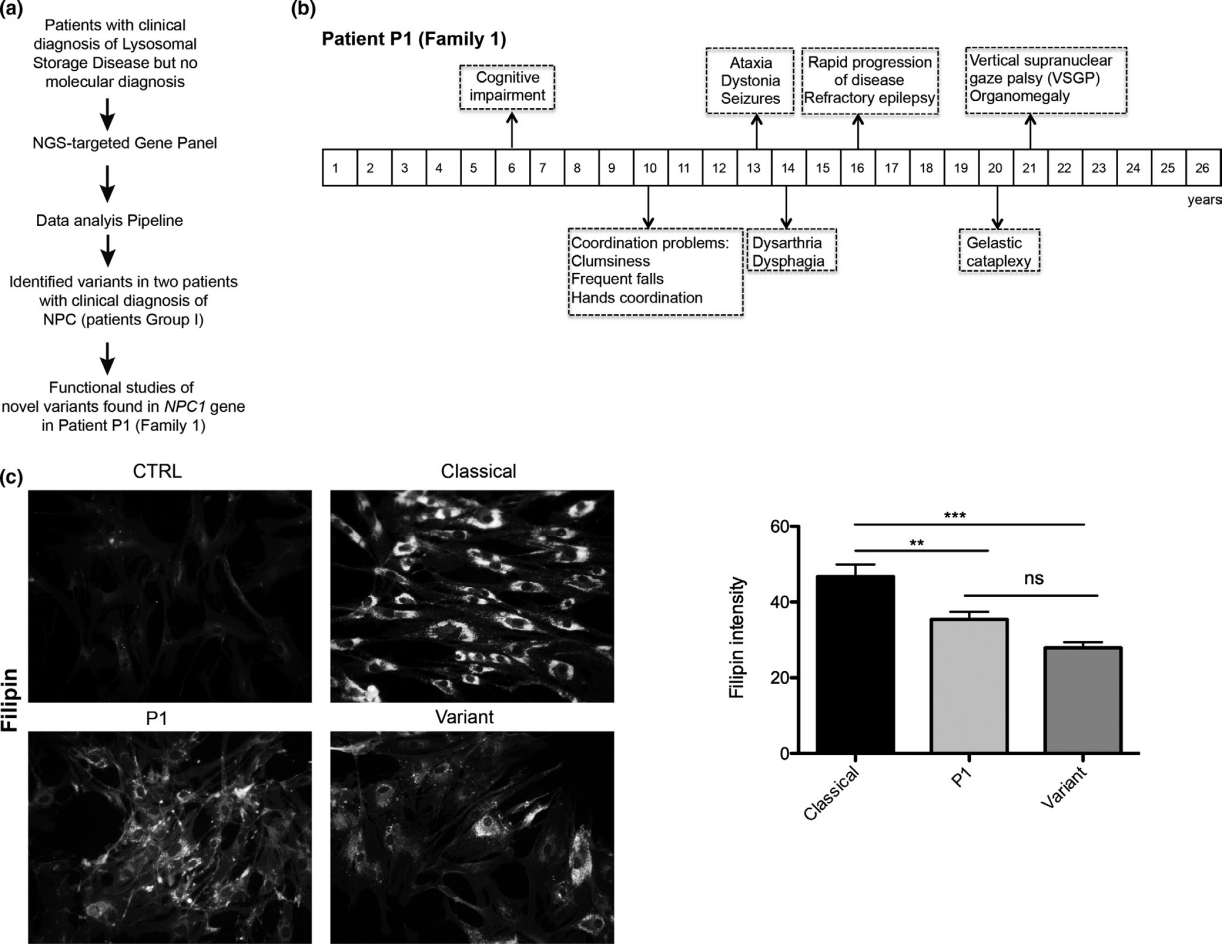
The NGS‐targeted gene panel allowed the screening of two NPC patients. One harbors known disease‐causing variants and for the second patient, a functional study was conducted. (a) Workflow of the study of the patients. (b) Clinical manifestation timeline for patient P1, with the first symptoms at 6 years old (juvenile form), cognitive impairment, ataxia, dystonia, and vertical supranuclear gaze palsy (VSGP) at 21 years old, compatible with an NPC diagnosis. (c) Unesterified cholesterol was labeled with filipin and staining intensity calculated from three independent experiments for patient P1, compared with a classical (patient P5) and variant (siblings P3/P3′). Data are mean ± SEM, ***p < 0.001, **p < 0.01 by ANOVA with Tukey's post hoc test. ns = not significant

In the present study, we investigated the molecular basis of disease in two unrelated NPC patients (one Portuguese, P1 and one Tunisian, P2), who were referred for analysis in an NGS‐targeted gene panel for LSD (Figure 1a). As a result, from the targeted sequencing workflow, we identified two heterozygous variants in the *NPC1* gene in each patient (Table 1, Group I): two known disease‐causing variants (c.7G=A; p.A3T and c.2882A=G; p.N961S) (Dvorakova et al., 2006; Run, Dolle, Schlitter, & Zschocke, 2008) in patient P2 and a novel missense variant (c.T1514G, p.V505G) in compound heterozygosity with a silent variant (c.1686G=A; p.V562V) in patient P1. The variants were confirmed by Sanger sequencing and shown to segregate within the families. No alterations/variants were detected in the *NPC2* gene. The novel variant p.V505G seemed to be very rare (only one heterozygous individual identified in the gnomAD database) and occurs in a conserved part of the protein. The silent variant p.V562V had previously been reported as a polymorphism in Spanish patients with clinical and biochemical diagnosis of NPC (Fernandez‐Valero et al., 2005) but is also absent in the Exome Aggregation Consortium and gnomAD databases. Therefore, additional studies were necessary to address the role of these variants in the disease pathogenicity.

Interestingly, when patient P1 fibroblasts were subjected to filipin staining, as a first approach biomarker to screen for NPC, the observed pattern was between a classical and variant profile, with a cholesterol accumulation less exuberant than a classical but slightly higher than the typical variant (quantified in Figure 1c). So, since P1 had a high clinical suspicion for NPC, as demonstrated in the clinical manifestation timeline (Figure 1b) and one positive biomarker (filipin staining), we decided to further investigate the novel *NPC1* variants.

This analysis also led us to revisit other Portuguese cases that had been molecularly screened for the *NPC1* gene but for whom it was not possible to establish a definitive diagnosis, as only one heterozygous pathogenic variant was detected. By doing so, we found a second patient with a clinical picture of NPC, who harbored the silent p.V562V in heterozygosity with a disease‐causing mutation: c.1552C=T (p.R518W) (Ribeiro et al., 2001) (patient referred to as K on the original reference). Therefore, we further enrolled her sample in the current study (P3), together with an affected sibling (P3′) and performed additional cDNA analysis to understand the molecular basis of disease in those patients as well.

In general, all NPC patients addressed in this study were clinically characterized as suffering from the juvenile form of the disease, according to the onset of neurological symptoms. A summary of the main clinical and biochemical features of the studied cases is presented in Table 1. For the full clinical description of patient P1 see Table S1.

### 3.2 The silent p.V562 V variant leads to the skipping of exon 11 and a premature stop codon

**FIGURE 2:**
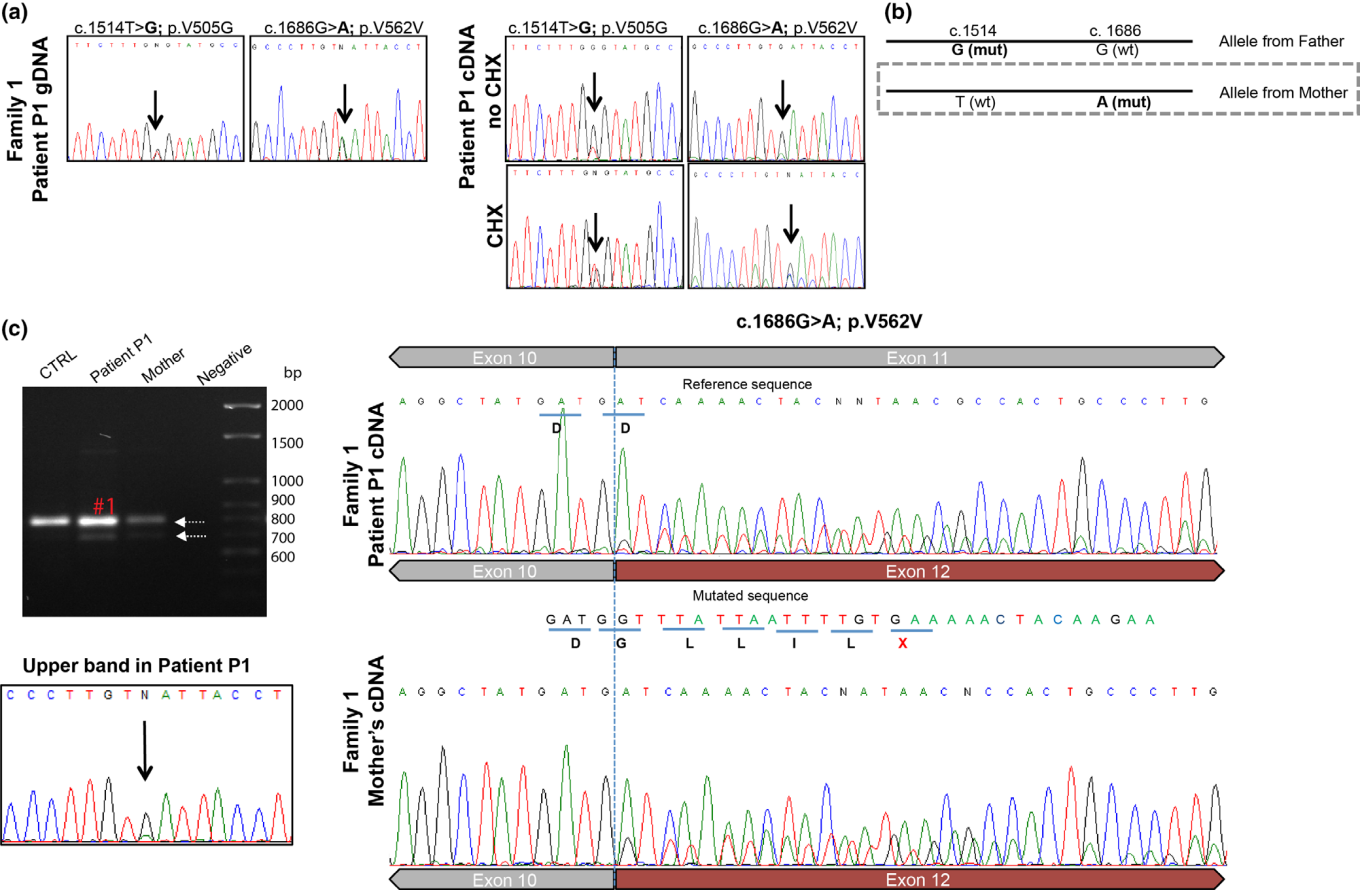
The silent variant p.V562V leads to the skipping of exon 11 and a premature stop codon (PTC). (a) The discrepancies between patient P1 gDNA (heterozygous for two nucleotide substitutions: c.1514T=G; p.V505G and c.1686G=A; p.V562V) and the cDNA (one allele more expressed than the other) led to the hypothesis of an unstable transcript. After segregation studies (chromatograms not shown) we observed that the allele inherited from the mother, that carries the variant c.1686G=A; p.V562V (b), is expressed in low abundance. Treatment with cycloheximide (CHX) partially stabilized the transcript (a, right and lower panel), suggesting NMD. (c) Agarose gel (ethidium‐bromide 1%) electrophoresis and sequencing chromatograms of cDNA‐derived PCR products (using PAXgene blood RNA) comprising exons 9‐13. The 697 bp amplified fragment represents the normal splice product and the 594 bp fragment represents the aberrant transcript, lacking exon 11. However, the upper band of 697 bp (corresponding to the normal length transcript) was sequenced separately (the band excised, purified, and sequenced) and it was shown that both the 1686G and 1686A alleles are present, suggesting that c.1686G=A; p.V562V is a leaky splicing variant. CTRL = control cDNA; negative = water control. At the boundaries of exon 10 and 11 it is possible to observe the second transcript, lacking exon 11 (c, right) and the PTC, marked in red. The same transcript was observed in the mother's cDNA (c, lower panel)

The analysis of gDNA by Sanger sequencing confirmed that patient P1 was heterozygous for both variants. Still, when we analyzed the patient's cDNA, one of the alleles seemed virtually absent, suggesting that one allele was more expressed than the other (Figure 2a). Segregation studies showed that the father was heterozygous for p.V505G, while the silent p.V562V had been inherited from the patient's mother. At the cDNA level, we could observe that the allele harboring the p.V562V generated a less abundant transcript (Figure 2a) and that observation raised the question of whether the p.V562V could lead to an unstable transcript most likely degraded by the nonsense‐mediated decay mechanism (NMD). Upon treatment of P1 primary fibroblasts with CHX for 5 h, on the assumption that inhibition of protein synthesis would prevent the rapid degradation of this mRNA (Carter et al., 1995), the unstable transcript became more abundant (Figure 2a, lower panel). In fact, at the boundary between exon 10 and 11, it was possible to visualize the presence of a second transcript lacking exon 11 in the sequencing electropherogram (Figure 2c). Coincidently, the same pattern was observed in the mother's cDNA (p.V562V carrier), as expected (Figure 2c, lower panel).

**FIGURE 3:**
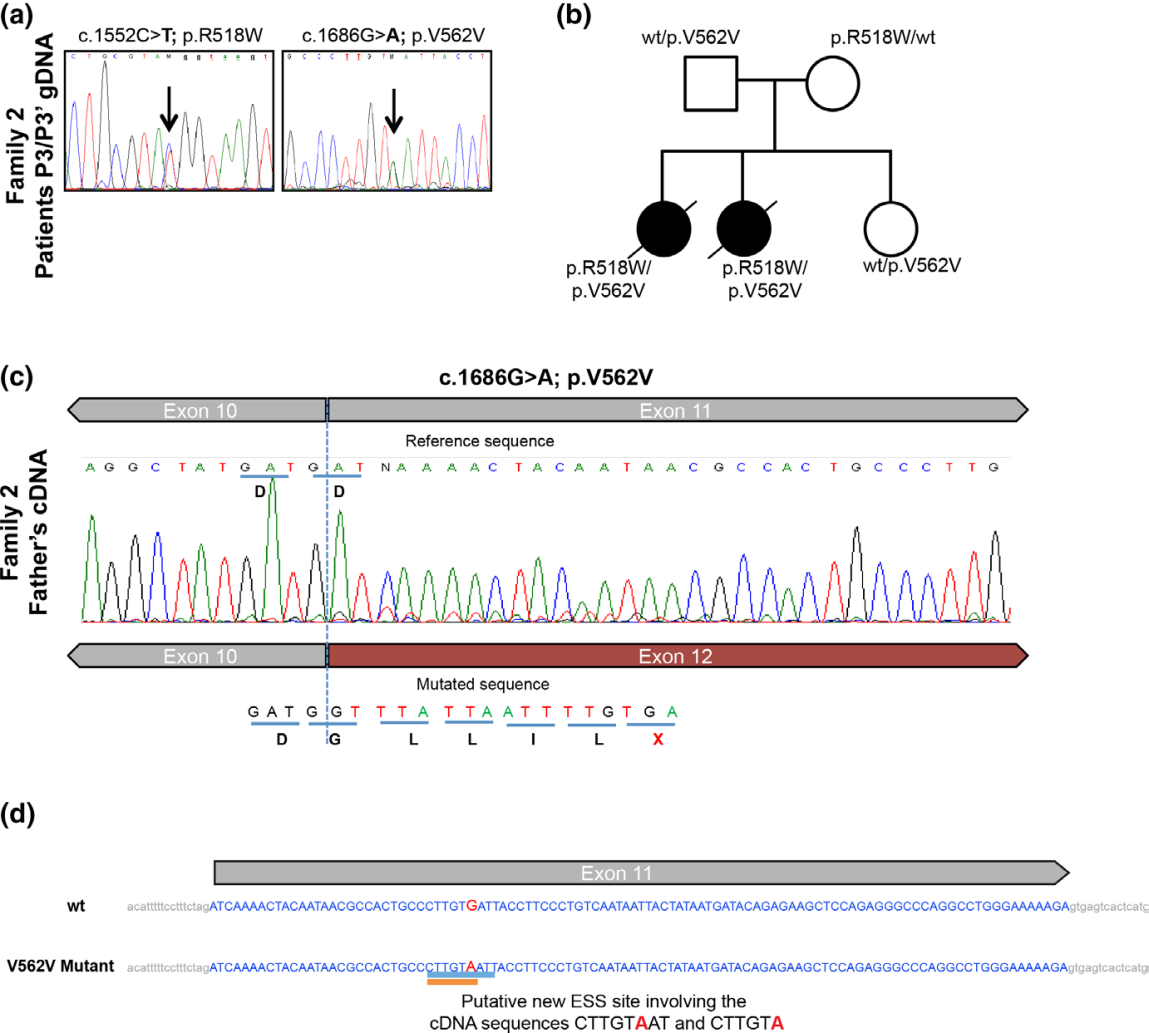
The silent variant p.V562 V was found in a second unrelated family (a) gDNA analysis of exons 9 and 11 in siblings P3 and P3′ (Family 2—F2), showing the variants in heterozygosity. (b) Subsequent/additional analysis on the parents and a non‐affected sister shows the segregation of the variants. (c) Sequencing chromatogram of cDNA‐derived PCR product comprising exons 9–13; at the boundaries of exon 10 and 11 it is possible to observe the second transcript, lacking exon 11 and with the premature stop codon. (d) Schematic representation of p.V562V localization on exon 11 and the effect on splicing based on *in silico* predictions (Human Splicing Finder—HSF and EX‐SKIP tools). EX‐SKIP compares the Exonic Splicing Enhancer (ESE)/Exonic Splicing Silencer (ESS) profile of a wild type (wt) and a mutated allele to determine if a specific exonic variant increases the chance of exon skipping. It calculates the total number of ESSs, ESEs, and their ratio. The V562V mutant is associated with a change in the ESS/ESE ratio which is compatible with a higher chance of exon skipping than in the wt allele. In addition, HSF (a tool to predict the effects of mutations on splicing signals or to identify splicing motifs in any human sequence) predicts that the V562V mutant leads to the creation of an ESS site. It involves the cDNA sequences CTTGTAAT (Zhang & Chasin, 2004) or CTTGTA (Goren et al., 2006), which might be associated with a potential alteration of splicing

This allowed us to establish the functional effect of the silent p.V562V, previously described as a polymorphism (Fernandez‐Valero et al., 2005) in the *NPC1* splicing process. The silent p.V562V mutation actually leads to exon 11 skipping, a frameshift and a PTC at amino acid 551 (the full‐length protein has 1278 amino acids). This observation is consistent with the results of the *in silico* analysis. The c.1686G=A; p.V562V variant occurs in the middle of exon 11 and according to Human Splicing Finder and EX‐SKIP predictions, creates a putative new exonic splicing silencer (ESS) binding site (Figure 3d). This is associated with a shift of 25% in the ESS/ESE ratio, which is compatible with a higher chance of exon skipping compared with the wt (Figure 3d). Interestingly, in the presence of this variant, the correctly spliced transcript is also produced, as shown by direct sequencing of the normal length transcript (Figure 2c, marked in red), where it is possible to observe both the 1686G and 1686A alleles. Also, the proportion of normal and aberrant spliced transcripts seems to be cell‐dependent, as differences were observed between blood and fibroblasts, the two cell types analyzed in the patient P1 (data not shown). It is reasonable to hypothesize that in other cell types, namely, in nerve cells, this ratio might also be different.

Having realized this, we then revisited other Portuguese families with a clinical diagnosis of NPC whose definitive molecular diagnosis had not been achieved since they presented with a sole *NPC1*‐causing variant in heterozygosity, assuming that some of them could harbor the silent p.V562V in the second allele and that this would fully explain the associated phenotype. In fact, the p.V562V variant was present in the second allele in two siblings from another unrelated family (F2:P3 and F2:P3′), as shown in Figure 3. Furthermore, in patient P3 the presence of the normally spliced transcript (also very minor) as a result of the p.V562V variant was observed.

Since our studies demonstrated that the silent p.V562V variant led to exon skipping, which gives rise to an aberrant transcript targeted by NMD, we propose the reclassification of this leaky splicing variant as disease‐causing and established the disease‐causing genotype for those siblings as p.R518W/p.V562V (Table 1).

### 3.3 Gene profiling of patient P1 (p.V505G/V562V) compared with NPC patients and controls showed upregulation of genes related to the UPR and ER stress

**FIGURE 4:**
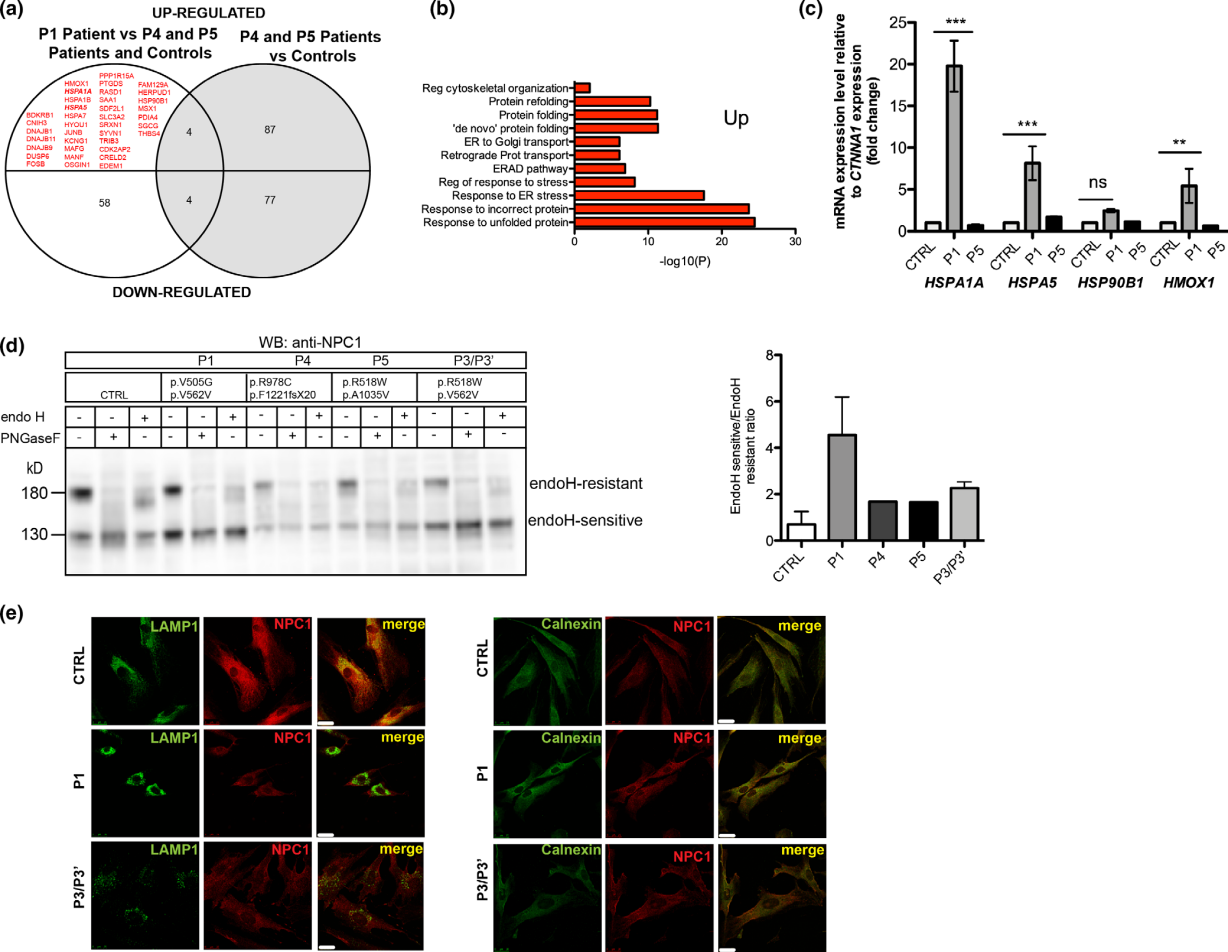
Patient P1 has differentially expressed genes (upregulated) associated with the UPR, but other NPC1 mutants exhibit delayed trafficking to lysosome. (a) List of genes with altered expression (*p* < 0.05, fold change =1.5) in P1 patient compared with P4, P5, and controls and P4 and P5 versus controls. Those that are upregulated exclusively in P1 are marked red. (b) After cluster analysis, the results showed that the most part of the genes are associated with ER stress and the UPR. (c) Relative expression levels of mRNA for *HSPA1A*, *HSPA5*, *HSP90B1*, *and HMOX1* genes (four of the obtained hits). Error bars represent standard deviation (SD) from three independent experiments. ***p < 0.001 P1 versus CTRL by one‐way ANOVA with Bonferroni's multiple comparison post hoc test (*n* = 3 in each group); ns = not significant. (d) CTRL and NPC1 primary human fibroblasts were treated with PNGaseF to deglycosylate the NPC1 protein (all N‐linked oligosaccharides) and Endo H, and subjected to Western blotting (WB) using anti‐NPC1 antibody. In CTRL the NPC1 protein is mostly Endo H‐resistant, but in all patients, the Endo H‐sensitive form is more abundant, suggesting that part of the protein was retained in the ER, and did not go for complex sugars formation, a process that only happens in the Golgi. Bands corresponding to complex glycosylated (Endo H‐resistant) and mannose rich forms (Endo H‐sensitive) were quantified and are displayed as Endo H‐sensitive/Endo H‐resistant ratio. (e) Immunostaining followed by confocal laser scanning microscopy of CTRL fibroblasts revealed that endogenous NPC‐1 protein colocalizes with LAMP1 and partially colocalizes with calnexin. In primary human fibroblasts of patient P1, NPC1 colocalizes much less with LAMP1. Instead, it presents a higher colocalization with calnexin. Scale bar 25 µm

To identify the gene expression profiles of the NPC patients versus controls, the expression pattern of more than 11,000 genes was analyzed. MARS‐Seq was performed on RNA samples isolated from cultured skin fibroblasts from controls and NPC patients (P1, P4, and P5—the last two had previously been diagnosed (Ribeiro et al., 2001)). After normalization of the counts, differential expression analysis and considering only genes with fold changes =1.5 and *p* < 0.05, we found up‐regulation of 43 genes and down‐regulation of 58 genes in P1, compared with P4, P5, and controls. Comparing P4 and P5 with controls, 87 genes were up‐regulated and 77 were down‐regulated (Figure 4a). We decided to focus on a group of clustered genes, which were exclusively up‐regulated in P1. Ingenuity pathway analysis (IPA) revealed that the genes differentially expressed in P1, compared with the other NPC patients and controls were associated with the following cellular pathways: response to unfolded protein (UPR); response to incorrect protein; response to ER stress; protein folding; protein refolding; ER‐associated degradation ERAD (Figure 4b). The most pronounced relative fold‐change was observed for the *HSPA5* (MIM #138120), *HSPA1A* (MIM #140550), and *HMOX1* (MIM #141250) genes, and confirmed by qtRT‐PCR (Figure 4c). The *HSPA5* and *HSPA1A* genes encode the chaperones BiP/GRP78 and Hsp72, respectively, both of which belong to the HSP70 family and *HMOX1* encodes heme oxygenase 1.

### 3.4 Intracellular processing and trafficking of NPC1 in the presence of the mutations under study

Next, we sought to determine the consequences of the identified variants at the cellular and biochemical levels. We analyzed the endogenous NPC1 protein using WB and IF. The anti‐NPC1 antibody detected two bands on the control, a major band at ~170 kDa and a minor one at ~130 kDa, which represent glycosylated and non‐glycosylated forms of the protein, respectively. After treatment with PNGase (which removes all N‐linked glycans), we observed a shift from the 170 kDa to 130 kDa (Figure 4d, lanes 1 and 2). Furthermore, we also applied endoglycosidase H (Endo H), which cleaves high‐mannose sugars and hybrid‐sugars (compatible with ER‐retained proteins) but not complex N‐linked sugars (proteins modified in Golgi and transported to lysosomes) to monitor trafficking of the NPC1 protein in the patients’ fibroblasts. We observed that, unlike the pattern observed for controls, there was a fraction of the protein that was retained in the ER (as observed in the band marked as Endo H‐resistant that is weaker in the patients’ samples—lanes 6, 9, 12, and 15). This pattern, which is significantly different from that observed in controls, was present in all analyzed patients, although the ratio of Endo H‐sensitive/‐resistant forms is higher in P1 than in P4 and P5, suggesting more ER retention of NPC1 in patient P1.

However, since all patients are compound heterozygous for *NPC1* mutations, the potential interaction between the two mutations in the NPC1 protein is difficult to assess. Interestingly, since the second mutation harbored by patient P1 is the silent mutation p.V562V, which leads to a truncated protein of ~55 kDa, the observed bands at ~170 kDa and ~130 kDa are most likely the result of the translation of only one *NPC1* allele: the one harboring the p.V505G mutation. This result further confirms the pathogenic nature of the p.V505G variant, namely, it impacts the NPC1 trafficking, leading to partial retention in the ER. Immunostaining followed by confocal laser microscopy of NPC1 in control cell lines showed that the wild type NPC1 localizes predominantly in lysosome, as confirmed by its colocalization with LAMP1 (Figure 4e, panel 1). Slight colocalization was also observed with the ER marker calnexin. In NPC1 mutants, nevertheless, no colocalization was found between NPC1 and LAMP1. This IF result corroborated the WB studies, confirming the ER retention of a fraction of the protein (Figure 4e). This partial retention happens in all studied cases, but is higher in P1, as the Endo H‐sensitive form at 130 kDa is more intense than the Endo H‐resistant form of 180 kDa, which could explain the upregulation of genes related to the UPR and ER stress.

## 4 DISCUSSION

The genomic sequencing of the *NPC1* and *NPC2* genes is recommended in order to confirm the diagnosis and is also the only method that supports the safe prenatal diagnosis. However, the molecular diagnosis can be complicated by a large number of sequence variants with unknown significance (VUS) and silent mutations (Probert et al., 2017; Tängemo, Weber, Theiss, Mengel, & Runz, 2011; Vanier et al., 2016). Therefore, the study of novel putative *NPC1* mutations at the protein and cellular levels is very important, specifically the effect on intracellular processing, trafficking, and localization of the NPC1 protein. In case of silent mutations, as the one reported here (p.V562V), cDNA analysis on the trio (index case and parents) is also essential to address the possible impact on the mRNA processing. Our study not only demonstrated that the p.V562V variant leads to exon 11 skipping generating an aberrant transcript, but also discovered evidence for leaky splicing control, most likely contributing to the intermediate biochemical phenotype of patient P1. This variant was found in three Portuguese patients (two siblings and one unrelated patient) all with a juvenile clinical manifestation. In the same protein region (luminal loop between TM2 and TM3) a second (novel) variant in compound heterozygosity was identified (p.V505G) in patient P1. Once we had successfully achieved the molecular diagnosis of these challenging cases (presence of silent mutations and VUS), and being aware that NPC disease is characterized by several different pathogenic cascades, we wanted to further understand the different pathomechanisms related to different *NPC1* mutations combinations but all leading to the juvenile type of the disease. For that, we used a single‐cell RNA‐seq approach (MARS‐Seq). It is known that NPC affects several organs and it will be interesting to analyze other cell types, but we were unable to compare gene expression profiles in cells other than cultured skin fibroblasts due to limited availability of other tissues. Interestingly, we found that P1 has massive upregulation of genes related to the UPR, namely *HSPA5*, *HSPA1A*, and *HMOX1* and a slight increase in *HSP90B1*. The ER stress has been described for the mutation p.I1061T since this mutated protein is retained in the ER (Schultz et al., 2018). In fact, immunoprecipitation of overexpressed and endogenous wild type and NPC1‐p.I1061T demonstrate interactions with the molecular chaperones Hsp70 (encoded by *HSPA1A*), Hsc70, Hsp90, and the ER‐localized chaperone calnexin (Schultz et al., 2016). Interestingly, the upregulation of genes encoding these chaperones is much higher in patient P1 than in P4 and P5, which deserves future study. Our results suggest that UPR activation changes among the different NPC patients (harboring different mutations), as previously described for Krabbe disease (Irahara‐Miyana et al., 2018). Since we observed UPR activation, our next research question was whether NPC1 mutants are retained in the ER or not. As recently described, there are several *NPC1* disease‐causing mutations leading to ER retention (Shammas et al., 2019) besides p.I1061T (Gelsthorpe et al., 2008; Nakasone et al., 2014). In the mutations studied herein, we observed a higher ratio of Endo H‐sensitive/Endo H‐resistant forms in P1 than in P4 and P5, suggesting than in P1 more NPC1 protein is retained in the ER, which could explain the upregulation of several UPR genes.

## CONFLICT OF INTERESTS

None declared.

## AUTHORS’ CONTRIBUTIONS

MTC, PC, and ELT phenotyped the patient P1. ME and SA processed and analyzed the NGS‐data. ME, MFC, and IR performed the Sanger sequencing. ME performed RNA extractions, cDNA synthesis, qtRT‐PCR, WB, and IF. MFC and JIS performed the CHX treatments, some RNA extractions, and cell culture maintenance. IR performed filipin staining of all patients. SMC performed and analyzed RNA‐seq. ME, MFC, IR, SA, and SMC analyzed and interpreted the data. SA, LV, DQ, LL, and AHF obtained funding support. ME wrote the first version of the manuscript and prepared the figures. MFC helped in the first version of the manuscript. SA coordinated the work and corrected the manuscript. All authors read the final version of the manuscript and gave their permission for publication.

## INFORMED CONSENT

Participants’ informed consent to the processing of their samples and the resulting data were a pre‐requisite.

## Supporting information

Table S1
