# Supplementary material for: NPC1 silent variant induces skipping of exon 11 (p.V562V) and unfolded protein response was found in a specific Niemann‐Pick type C patient
Source: Mol Genet Genomic Med. 2020 Sep 15;8(11):e1451. doi: 10.1002/mgg3.1451 (PMC7667330; doi:10.1002/mgg3.1451)
Supplement: Supplementary file 1 — Table S1 [file MGG3-8-e1451-s001.docx]

***NPC1* silent variant induces complete skipping of exon 11 (p.V562V) and unfold protein response was found in a specific Niemann-Pick type C patient**

**Running title: *NPC1* silent variant (p.V562V) and unfold protein response**

Marisa Encarnação^1,2,3^, Maria Francisca Coutinho^1,3^, Soo Min Cho^4^, Maria Teresa Cardoso^5^, Isaura Ribeiro^6,7,8^, Paulo Chaves^5^, Juliana Inês Santos^1^, Dulce Quelhas^6,7,8^, Lúcia Lacerda^6,7,8^, Elisa Leão Teles^5^, Anthony H Futerman^4^, Laura Vilarinho^1,2,3^, and Sandra Alves^1,3^

^1^Research & Development Unit, Human Genetics Department, National Institute of Health Doutor Ricardo Jorge, Porto - Portugal

^2^Newborn Screening, Metabolism & Genetics Unit, Human Genetics Department, National Institute of Health Doutor Ricardo Jorge, Porto - Portugal

^3^Center for the Study of Animal Science, CECA-ICETA, University of Porto - Portugal

^4^ Department of Biomolecular Sciences, Weizmann Institute of Science, Rehovot – Israel

*^5^Centro de Referência de Doenças Metabólicas do Centro Hospitalar Universitário São João*, Porto - Portugal

^6^*Unidade de Bioquímica Genética, Centro de Genética Médica Jacinto Magalhães- Centro Hospitalar e Universitário do Porto (CHP)*, Porto - Portugal

^7^Clinical and Experimental Human Genomics group (CEHG), UMIB-Unit for Multidisciplinary Research in Biomedicine, ICBAS, University of Porto, Porto - Portugal

^8^MetabERN-European Reference Network for Rare Hereditary Metabolic Disorder, Reference Centre for Diagnosis and Treatment- CHP, Porto - Portugal

Corresponding author:

Sandra Alves

Research and Development Unit, Department of Human Genetics, National Institute of Health Doutor Ricardo Jorge

Rua Alexandre Herculano, 321

4000-055 Porto, Portugal

Tel.: +351 223 401 113; Fax: (+351) 223 401 109

E-mail: sandra.alves@insa.min-saude.pt; alvessandra@hotmail.com

**Table S1** Detailed clinical description of the patient P1 with the age at onset of the visceral, neurological and psychiatric symptoms.

| Genotype | | p.Val505Gly/Val562Val |
| --- | --- | --- |
| Gender | | Male |
| Age of presentation | | 12 years |
| First symptoms | | 6 years |
| Magnetic Resonance Imaging (MRI) | | 17, 20 , 22 years |
| **Visceral** | Splenomegaly | 21 years |
|  | Hepatomegaly | 21 years |
|  | Neonatal cholestatic jaundice | No |
|  | Hydrops foetalis | No |
|  | Pneumopathologies | No |
|  | Mild thrombocytopenia | No |
| **Neurological** | VSGP | 21 years |
|  | Gelastic cataplexy | 20 years |
|  | Ataxia | 13 years |
|  | Dystonia | 13 years |
|  | Dysarthria | 14 years |
|  | Dysphagia | 14 years |
|  | Hypotonia | No |
|  | Clumsiness | 10 years |
|  | Delayed developmental milestones | 6 years |
|  | Seizures | 13 years |
|  | Hearing loss | No |
| **Psychiatric** | Developmental delay and pre-senile cognitive decline | 6 years |
|  | Organic psychosis | No |
|  | Disruptive/agressive behaviour | No |
|  | Progressive developmental of treatment-resistant psychiatric symptoms | 12 years |
